# Long-Term Deutetrabenazine Treatment for Tardive Dyskinesia Is Associated With Sustained Benefits and Safety: A 3-Year, Open-Label Extension Study

**DOI:** 10.3389/fneur.2022.773999

**Published:** 2022-02-23

**Authors:** Robert A. Hauser, Hadas Barkay, Hubert H. Fernandez, Stewart A. Factor, Joohi Jimenez-Shahed, Nicholas Gross, Leslie Marinelli, Amanda Wilhelm, Jessica Alexander, Mark Forrest Gordon, Juha-Matti Savola, Karen E. Anderson

**Affiliations:** ^1^University of South Florida Parkinson's Disease and Movement Disorders Center, Tampa, FL, United States; ^2^Teva Pharmaceuticals, Netanya, Israel; ^3^Cleveland Clinic, Cleveland, OH, United States; ^4^Emory University, Atlanta, GA, United States; ^5^Icahn School of Medicine at Mount Sinai, New York, NY, United States; ^6^Teva Pharmaceuticals, West Chester, PA, United States; ^7^Teva Pharmaceuticals, Basel, Switzerland; ^8^Georgetown University, Washington, DC, United States

**Keywords:** deutetrabenazine, efficacy, safety, tardive dyskinesia, treatment

## Abstract

**Background:**

Deutetrabenazine is a vesicular monoamine transporter 2 inhibitor approved for the treatment of tardive dyskinesia (TD) in adults. In two 12-week pivotal studies, deutetrabenazine demonstrated statistically significant improvements in Abnormal Involuntary Movement Scale (AIMS) scores, with favorable safety/tolerability in TD patients. This study reports long-term efficacy and safety of deutetrabenazine in a 3-year, single-arm, open-label extension (OLE) study.

**Methods:**

Patients who completed the pivotal studies could enroll in this single-arm OLE study, titrating up to 48 mg/day based on dyskinesia control and tolerability. Efficacy was assessed based on change from baseline in total motor AIMS score, Clinical Global Impression of Change (CGIC) and Patient Global Impression of Change (PGIC), and quality of life (QOL) assessments. Safety evaluation included adverse event (AE) incidence, reported using exposure-adjusted incidence rates, and safety scales.

**Results:**

343 patients enrolled in the study (6 patients were excluded). At Week 145 (mean dose: 39.4 ± 0.83 mg/day), mean ± SE change from baseline in total motor AIMS score was −6.6 ± 0.37 and 67% of patients achieved ≥50% improvement in total motor AIMS score. Based on CGIC and PGIC, 73% and 63% of patients achieved treatment success, respectively. QOL improvements were also observed. Deutetrabenazine was generally well tolerated, with low rates of mild-to-moderate AEs and no new safety signals; most safety scales remained unchanged over time.

**Conclusions:**

Long-term deutetrabenazine treatment was associated with sustained improvement in AIMS scores, indicative of clinically meaningful long-term benefit, and was generally well tolerated. Results suggest deutetrabenazine may provide increasing benefit over time without increases in dose.

## Introduction

Tardive dyskinesia (TD) is a hyperkinetic movement disorder resulting from chronic exposure to dopamine D2 receptor antagonists (DRAs), including antipsychotics and antiemetics (1, 2). The mechanism of TD is unclear; however, dopamine receptor blockade in the nigrostriatal pathway may lead to upregulation of dopamine D2 receptors resulting in hypersensitivity and higher affinity for dopamine (3). This persistent hyperdopaminergic state can lead to downstream changes that interrupt normal mechanisms controlling movement (3, 4). TD is characterized by involuntary movements, such as stereotypy, chorea, dystonia, tics, tremor, or a combination of these, as well as potential sensory symptoms (4–6). TD is a socially stigmatizing disease that has been linked to poor quality of life and social withdrawal, as well as increased morbidity and mortality (6–9). The estimated prevalence of TD is ~15% to 40% in the United States for those exposed to DRAs (5, 10–13).

If TD prevention cannot be achieved by avoiding or limiting DRA use, vesicular monoamine transporter 2 (VMAT2) inhibitors are the recommended first-line therapy, with Level A evidence supporting their use (3, 14). Deutetrabenazine is a novel, highly selective VMAT2 inhibitor approved by the US Food and Drug Administration (FDA) for the treatment of chorea associated with Huntington's disease and TD (15). Deutetrabenazine contains deuterium, a naturally occurring, nontoxic form of hydrogen that attenuates drug metabolism and improves tolerability by reducing adverse events (AEs) related to peak concentrations (16–18). The major circulating metabolites (α-dihydrodeutetrabenazine [α-HTBZ] and β-HTBZ) of deutetrabenazine are reversible inhibitors of VMAT2, resulting in decreased uptake of monoamines into synaptic vesicles and depletion of monoamine stores, the latter due to metabolism by monoamine oxidase. Reducing dopamine release may decrease the overstimulation of supersensitive D2 dopamine receptors in the motor striatum that causes TD (19). The therapeutic effect is presumed to be related to rebalancing the direct and indirect pathways of the cortico-striatal-thalamic circuit (15, 18, 20–22).

In two pivotal, 12-week, phase 3 clinical trials (ARM-TD and AIM-TD) in patients with TD, deutetrabenazine was associated with clinically significant improvements in Abnormal Involuntary Movement Scale (AIMS) scores compared to placebo, with a favorable safety profile (16, 23). Patients who completed either ARM-TD or AIM-TD were eligible to enroll in a single-arm open-label extension (OLE) study designed to evaluate the long-term efficacy and safety of deutetrabenazine in patients with TD; 2-year results were previously reported (24). Here, we report the long-term efficacy and safety of deutetrabenazine treatment in patients with TD who were treated for up to 3 years.

## Methods

### Study Design

The OLE was a 3-year, single-arm study of deutetrabenazine in patients with moderate to severe TD (ClinicalTrials.gov Identifier: NCT02198794) that was conducted at 76 centers in the United States and Europe from October 15, 2014 to October 16, 2019 (Supplementary Figure 1). The study was divided into Parts A, B, and C. Part A included a 6-week titration period and a long-term (up to 3-year) treatment period. Part B included a 1-week, double-blind, randomized withdrawal period and a 12-week, open-label treatment period after completion of the randomized withdrawal. Part C, offered in European Union countries only, consisted of a 52-week period of continued open-label treatment with reduced safety assessments. Here, we report results from Part A, which included a 4-week, post-treatment, follow-up period for patients who terminated the study early or who did not enroll in Part B. Results of Parts B and C will be reported separately.

Following the 1-week washout from the parent study's treatment (deutetrabenazine or placebo), all patients were initiated on deutetrabenazine 12 mg/day, regardless of prior treatment arm, and re-titrated. During the 6-week titration period, the dose of deutetrabenazine was increased on a weekly basis in a response-driven manner at an interval of 6 mg/day until (1) adequate dyskinesia control was achieved, (2) a clinically significant AE occurred, or (3) the maximum allowable dose was reached. Based on population pharmacokinetic analyses (25), for patients with a body weight <100 kg, the maximum total daily dose of deutetrabenazine allowed was 48 mg/day, unless the patient was on a strong CYP2D6 inhibitor, in which case the maximum daily dose allowed was 36 mg/day (75% of the maximum total daily dose). For patients with body weight of ≥100 kg, the maximum total daily dose of deutetrabenazine allowed was 60 mg/day, unless the patient was on a strong CYP2D6 inhibitor, in which case the maximum daily dose allowed was 42 mg/day (~75% of the maximum total daily dose of 60 mg, as the 6-mg tablet was the smallest mg increment available). Further dose adjustments were permitted on a weekly basis to optimize dyskinesia control or minimize AEs.

### Patients

Patients were eligible to enroll in the OLE study if they were aged ≥18 years and had successfully completed a parent study (ARM-TD or AIM-TD). Prior to screening in the parent studies, eligible patients had a history of DRA use for ≥3 months (or 1 month in patients aged ≥60 years) and a clinical diagnosis of TD with symptoms occurring for ≥3 months. Patients with underlying psychiatric illness were included if they were psychiatrically stable with no change in psychoactive medications for ≥30 days prior to screening in the parent studies (≥45 days for antidepressants) or were on a stable therapy of long-acting depot medications lasting ≥3 months before screening in the parent studies. Full eligibility criteria are shown in Supplementary Table 1. Successful completion of a parent study was defined as (1) study participation through Week 13; (2) the patient had generally been compliant with study drug administration and procedures, in the opinion of the investigator; and (3) the patient had no ongoing AEs that were serious, severe in intensity, or expected to interfere with their participation in this study.

This study was conducted in full accordance with the International Council for Harmonisation Good Clinical Practice Consolidated Guideline (E6) and any applicable national and local laws and regulations. The protocol was submitted to the independent ethics committee or institutional review board according to national or local regulations. Each patient provided written informed consent prior to the study.

### Efficacy Assessments

Efficacy was evaluated based on change from baseline in total motor AIMS score (items 1–7, evaluating orofacial movements [items 1–4] and extremity and truncal movements [items 5–7] on a 5-point anchored scale), as assessed by local site rating; percentage of patients achieving ≥50 or ≥70% improvement in total motor AIMS score from baseline; percentage of patients achieving a sustained response, defined as the percentage of patients achieving ≥50% improvement in AIMS score at Week 15 who continued this improvement at Weeks 54, 106, or 145; percentage of patients achieving treatment success, defined as “much improved” or “very much improved,” based on physician assessment on the Clinical Global Impression of Change (CGIC); percentage of patients achieving treatment success based on patient assessment on the Patient Global Impression of Change (PGIC); change from baseline in modified Craniocervical Dystonia Questionnaire (mCDQ-24) score assessing quality of life, measured through Week 106 (Week 106 was the last site visit for these data to be obtained); and change from baseline in AIMS items 8, 9, and 10 (clinician-rated global judgments of the overall severity of abnormal movements, incapacitation due to abnormal movements, and patient's awareness of abnormal movements, respectively) through Week 145.

### Safety Assessments

Safety was evaluated based on AEs, including serious AEs, treatment-related AEs, AEs leading to withdrawal, AEs leading to dose reduction, and AEs leading to dose suspension. AEs were assessed separately for the titration, maintenance, and overall treatment and follow-up periods. Observed values and changes from baseline were assessed for clinical laboratory parameters (e.g., hematology, chemistry, and urinalysis) and vital signs, as well as for safety scales including the Unified Parkinson's Disease Rating Scale (UPDRS) Part III (motor examination), Barnes Akathisia Rating Scale (BARS), Hospital Anxiety and Depression Scale (HADS), Columbia–Suicide Severity Rating Scale (C-SSRS), Epworth Sleepiness Scale (ESS), and Montréal Cognitive Assessment (MoCA). Observed values in electrocardiogram (ECG) parameters and shifts from screening for clinically significant abnormal findings were also assessed, including the incidence of patients having an on-treatment Fridericia's corrected QT interval (QTcF) >450 ms, >480 ms, or >500 ms, or a change from baseline in QTcF >30 ms or >60 ms.

### Statistical Analysis

Efficacy and safety analyses were performed on the intent-to-treat population, which included all enrolled patients; all patients who received ≥1 dose study drug. Descriptive statistics of the observed data were used to summarize all continuous and categorical variables. There was no imputation of missing data. AEs were assessed via exposure-adjusted incidence rates (EAIRs), which were calculated as the number of patients per patient-year. In each AE category, patients with an AE contributed treatment exposure up to the day of their first AE, and patients without an AE contributed their entire treatment exposure.

## Results

### Patients

A total of 343 patients were enrolled in the study, with 1 site (6 patients) excluded from all analyses due to site data integrity issues as reported to the FDA. Of the 337 patients included in this analysis, 227 patients previously received deutetrabenazine and 110 patients previously received placebo in the parent studies. Patient disposition is presented for Part A only (Supplementary Figure 2).

Overall, 163 (48%) patients discontinued from the study during the 3 years of open-label treatment; 88 (26%) patients discontinued in the first year and 44 (18%) patients discontinued in the second year (Supplementary Table 2). Reasons for discontinuation included AE (41 [12%] patients), withdrawal by subject (79 [23%] patients), lost to follow-up (24 [7%] patients), lack of efficacy (9 [3%] patients), other reasons (5 [1%] patients), noncompliance with study drug (3 [<1%] patients), protocol deviation (1 [<1%] patient), and study terminated (1 [<1%] patient).

Demographic and clinical characteristics from baseline of the parent studies are shown in Supplementary Table 3. The mean (standard deviation [SD]) time since TD diagnosis was 5.72 (5.75) years at baseline of the parent studies; mean (SD) total motor AIMS score was 10.7 (4.68) at baseline of the OLE study. At baseline of the parent studies, 61% of patients reported a psychotic disorder, 34% reported a mood disorder, and 75% of patients were using a DRA. Of 163 patients with available data at Week 145, 107 (66%) patients were using a DRA.

### Dosing

**Figure 1:**
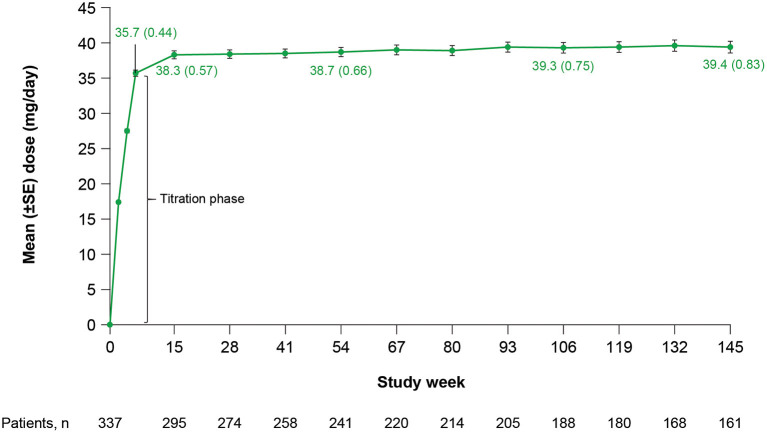
Total daily deutetrabenazine dose over time. SE, standard error.

Mean (standard error [SE]) total daily dose was 35.7 (0.44) mg/day at Week 6 (end of titration) and 38.3 (0.57) mg/day at Week 15, and remained stable throughout the rest of the study (Figure 1). Doses ranged from 12 to 42 mg/day at Week 6 and 12 to 48 mg/day at Week 15. At Week 6 (end of titration period; *n* = 318), 21 (7%) patients received a deutetrabenazine dose <24 mg/day, 149 (47%) patients received a dose between 24 and 36 mg/day, and 148 (47%) patients received a dose between >36 and 48 mg/day. At Week 145 (*n* = 161), 9 (6%) patients received a dose <24 mg/day, 64 (40%) patients received a dose between 24 and 36 mg/day, 83 (52%) patients received a dose between >36 and 48 mg/day, and 5 (3%) patients received a dose >48 mg/day.

### Efficacy

**Figure 2:**
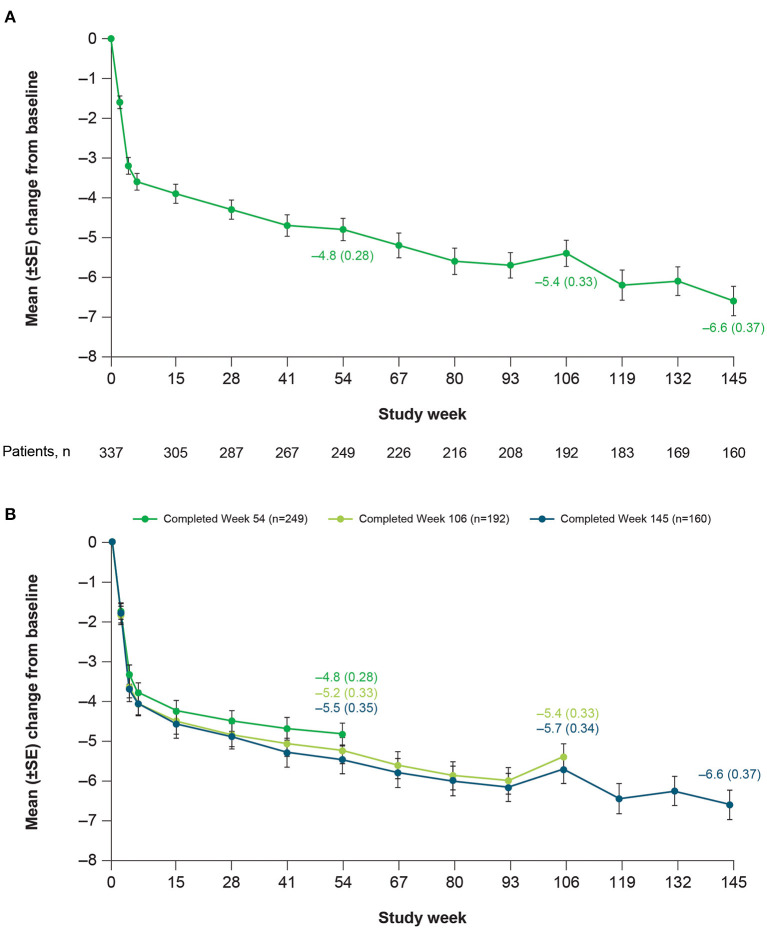
Mean change in total motor AIMS score items (1–7) over time in **(A)** the overall population and **(B)** among completers at Weeks 54, 106, and 145. AIMS, Abnormal Involuntary Movement Scale; SE, standard error.

Patients receiving deutetrabenazine experienced continued improvements in total motor AIMS score that were sustained over 3 years, with minimal changes in dose over time (Figure 2A). Mean change (SE) in AIMS score from baseline of the OLE study was −3.6 (0.21) at the end of titration (Week 6), −3.9 (0.24) at Week 15, −4.8 (0.28) at Week 54, −5.4 (0.33) at Week 106, and −6.6 (0.37) at Week 145. To help assess whether the observed increasing efficacy over time was driven by selection bias (i.e., patients who did not respond or who responded less preferentially dropped out of the study over time), *post-hoc* analyses were conducted in patients who completed Weeks 54, 106, and 145. Results indicated that the improvement in AIMS score was not derived from early terminations, with mean (SE) changes from baseline in total motor AIMS score at Week 54 of −4.8 (0.28), −5.2 (0.33), and −5.5 (0.35) for patients who completed 54, 106, and 145 weeks of treatment, respectively (Figure 2B). Notably, within each of these completer groups, increasing efficacy over time was observed.

**Figure 3:**
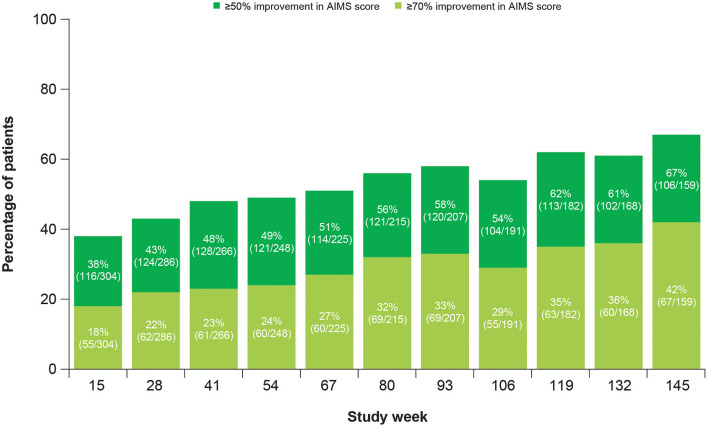
Percentage of patients achieving improvement in AIMS score from baseline. AIMS, Abnormal Involuntary Movement Scale.

**Figure 4:**
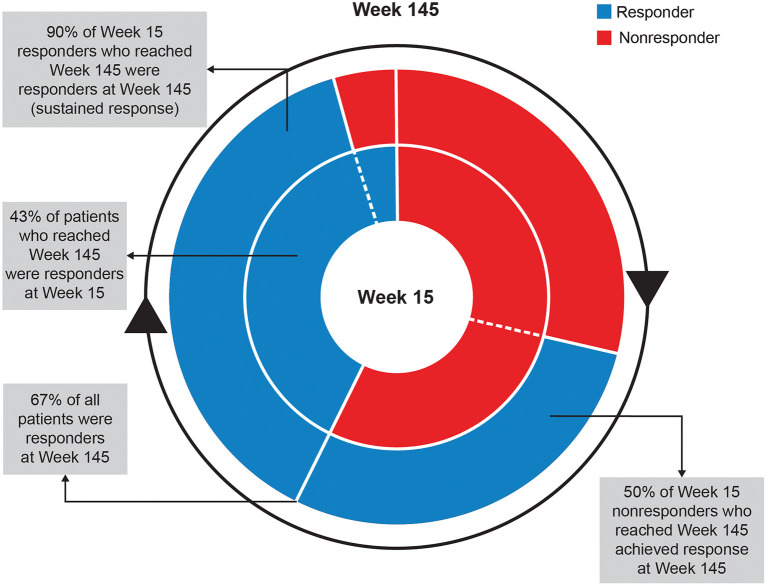
Sustained response analysis of ≥50% of AIMS responders at Weeks 15 and 145. AIMS, Abnormal Involuntary Movement Scale.

At Week 145, 67% of patients achieved ≥50% improvement and 42% of patients achieved ≥70% improvement in total motor AIMS score from baseline (Figure 3). For patients who achieved ≥50% improvement in total motor AIMS score from baseline, the mean (SE) total daily dose was 37.6 (0.94) mg/day at Week 54, 38.1 (1.06) mg/day at Week 106, and 39.0 (1.07) mg/day at Week 145, indicating improvements in symptoms with stable dosing over time. Of those patients who achieved ≥50% improvement in total motor AIMS score at Week 15 and reached Week 145, 89.7% sustained their response at Week 145 (Figure 4).

**Figure 5:**
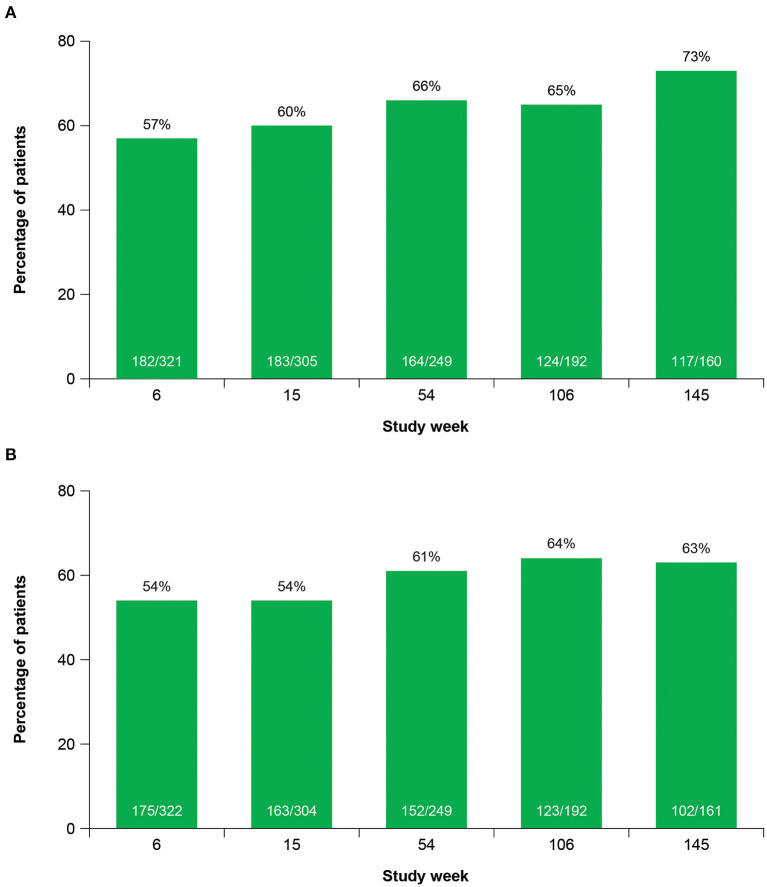
Treatment success^a^ over time on the **(A)** CGIC and **(B)** PGIC. CGIC, Clinical Global Impression of Change; PGIC, Patient Global Impression of Change. ^a^Treatment success was defined as “very much improved” or “much improved.”

**Figure 6:**
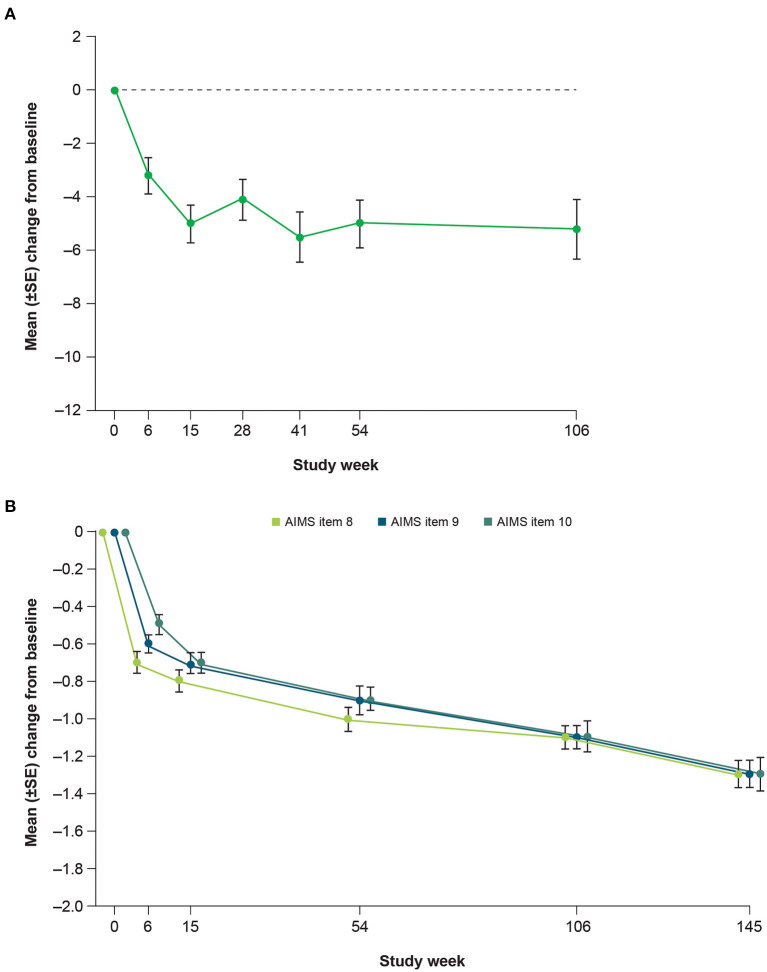
Mean change from baseline in **(A)** mCDQ-24 total score^a^ and **(B)** AIMS items 8 through 10 over time. mCDQ-24, modified Craniocervical Dystonia Questionnaire; AIMS, Abnormal Involuntary Movement Scale; SE, standard error. ^a^Week 106 was the last site visit for mCDQ-24 data to be obtained.

Clinicians and patients generally recognized improvement in TD symptoms with deutetrabenazine treatment based on assessments of CGIC and PGIC over time. From Week 15 to Week 145, the proportion of patients achieving treatment success on the CGIC increased from 60 to 73%, and the proportion of patients achieving treatment success on the PGIC increased from 54 to 63% (Figure 5). Clinically meaningful improvements in quality of life were observed based on patient responses to the mCDQ-24, which was assessed through Week 106 (Figure 6A). At Week 106, mean change (SE) from baseline in mCDQ-24 total score was −5.2 (1.11), with improvements seen across the stigma, emotional, pain, activities of daily living, and social subdomains (Figure 6A and Supplementary Figure 3). Through Week 145, patients experienced sustained improvements in AIMS items 8, 9, and 10, which assess clinician-rated global judgments of the overall severity of abnormal movements, incapacitation due to abnormal movements, and patient's awareness of abnormal movements, respectively (Figure 6B).

### Safety

Over a mean (SE) of 106.5 (3.20) weeks of treatment (up to 158 weeks for safety assessments) and 688 patient-years of exposure, deutetrabenazine was generally safe and well tolerated. The EAIR for patients who experienced any AE was 1.24 patients per patient-year (Table 1). The EAIRs for severe AEs, treatment-related AEs; serious AEs; and AEs leading to discontinuation, dose suspension, and dose reduction were generally low. EAIRs for AEs of interest, including depression, parkinsonism, and torsade de pointes/QT prolongation, were low (Table 1). The most commonly reported AEs were anxiety, depression, somnolence, decreased weight, and urinary tract infection (Table 2). In general, the incidence of these AEs was lower during the titration vs. the maintenance period, which is expected given the longer duration of the maintenance period. Exceptions with a numerically higher incidence during the titration vs. maintenance period included headache (4% vs. 3%) and fatigue (3% vs. 2%). The most common abnormal ECG findings were QTcF >450 ms (30 [9%] patients) and change in QTcF >30 ms (45 [13%] patients; Supplementary Table 4). Eight deaths were reported during the study; none were considered to be related to study drug by the investigator.

**Table 1:** EAIRs of Patients With AEs^a^.

**Category**	**Number of patients/patient-year (EAIR)**
AEs	268/215.8 (1.24)
Severe AEs	55/629.9 (0.09)
Treatment-related AEs	153/428.5 (0.36)
Serious AEs	67/617.6 (0.11)
AEs leading to discontinuation	41/683.3 (0.06)
AEs leading to dose suspension	29/657.7 (0.04)
AEs leading to dose reduction	53/572.4 (0.09)
Deaths, *n*	8
**AEs of interest**	
Torsade de pointes/QT prolongation (SMQ)^b^	8/679.2 (0.01)
Akathisia (SMQ)^c^	10/675.2 (0.01)
Suicide/self-injury (SMQ)^d^	14/673.1 (0.02)
Somnolence and sedation (PT)^e^	41/621.3 (0.07)
Parkinson-like events (SMQ)^f^	51/606.1 (0.08)
Parkinsonism (PT)^g^	7/678.2 (0.01)
Depression (SMQ)^h^	50/611.5 (0.08)

*EAIR, exposure-adjusted incidence rate; AE, adverse event; SMQ, standardized MedDRA query; PT, preferred term*.

a *EAIRs were calculated as the number of patients per patient-year; in each category, patients with an AE contributed treatment exposure up to the day of their first AE, and patients without an AE contributed their entire treatment exposure. AEs summarized are those that began or worsened after treatment with study drug*.

b *SMQ for torsade de pointes/QT prolongation (narrow and broad search) included cardiac arrest, cardiac death, cardiac fibrillation, cardio-respiratory arrest, electrocardiogram QT interval abnormal, electrocardiogram QT prolonged, electrocardiogram U-wave abnormality, electrocardiogram repolarization abnormality, long QT syndrome, long QT syndrome congenital, loss of consciousness, sudden cardiac death, sudden death, syncope, torsade de pointes, ventricular arrhythmia, ventricular fibrillation, ventricular flutter, ventricular tachyarrhythmia, and ventricular tachycardia*.

c *SMQ for akathisia (narrow and broad search) included akathisia, extrapyramidal disorder, hyperkinesia, hyperkinesia neonatal, motor dysfunction, movement disorder, psychomotor hyperactivity, and restlessness*.

d *SMQ for suicide/self-injury (narrow search) included completed suicide, depression suicidal, intentional overdose, intentional self-injury, poisoning deliberate, self-injurious behavior, self-injurious ideation, suicidal behavior, suicidal ideation, and suicide attempt*.

e *PTs for somnolence and sedation included sedation, somnolence, sopor, stupor, and lethargy*.

f *SMQ for Parkinson-like events (narrow and broad search) included action tremor, akinesia, bradykinesia, bradyphrenia, cogwheel rigidity, drooling, dysphonia, extrapyramidal disorder, freezing phenomenon, gait disturbance, hypertonia, hypertonia neonatal, hypokinesia, hypokinesia neonatal, masked facies, micrographia, mobility decreased, motor dysfunction, movement disorder, muscle rigidity, muscle tone disorder, musculoskeletal stiffness, on and off phenomenon, Parkinson's disease, Parkinson's disease psychosis, parkinsonian crisis, parkinsonian gait, parkinsonism rest tremor, parkinsonism, parkinsonism hyperpyrexia syndrome, postural reflex impairment, postural tremor, resting tremor, tremor, tremor neonatal, and walking disability*.

g *PT for parkinsonism was included in the SMQ for Parkinson-like events*.

h *SMQ for depression (excluding suicide/self-injury; narrow search) included activation syndrome, adjustment disorder with depressed mood, adjustment disorder with mixed anxiety and depressed mood, agitated depression, anhedonia, antidepressant therapy, childhood depression, decreased interest, depressed mood, depression, depression postoperative, depressive symptom, dysphoria, electroconvulsive therapy, feeling guilty, feeling of despair, feelings of worthlessness, helplessness, major depression, menopausal depression, poststroke depression, postictal depression, and postpartum depression*.

**Table 2:** Most Common (≥4%) EAIRs^a^.

**AE**	**Number of patients/patient-year (EAIR)**
Anxiety	42/636.0 (0.07)
Somnolence	34/629.7 (0.05)
Depression	33/640.1 (0.05)
Weight decreased	30/658.5 (0.05)
Urinary tract infection	29/642.5 (0.05)
Diarrhea	27/646.5 (0.04)
Headache	23/644.3 (0.04)
Hypertension	23/652.7 (0.04)
Nasopharyngitis	20/651.7 (0.03)
Dyskinesia	18/664.0 (0.03)
Fall	17/663.6 (0.03)
Fatigue	16/657.1 (0.02)
Influenza	16/672.6 (0.02)
Bradykinesia	15/656.4 (0.02)
Nausea	15/670.0 (0.02)
Weight increased	15/670.7 (0.02)
Back pain	14/665.6 (0.02)
Depressed mood	14/663.3 (0.02)
Dizziness	14/667.3 (0.02)
Insomnia	14/659.7 (0.02)
Tremor	14/670.5 (0.02)

*EAIR, exposure-adjusted incidence rate; AE, adverse event*.

a *Calculated as the number of patients per patient-year; in each category, patients with an AE contributed treatment exposure up to the day of their first AE, and patients without an AE contributed their entire treatment exposure*.

A total of 53 (16%) patients had an AE leading to dose reduction, most of which were mild to moderate in severity and were determined by the investigator to be possibly or probably related to treatment. The most common AEs leading to dose reduction were bradykinesia (13 [4%] patients); somnolence (11 [3%] patients); parkinsonism (5 [1%] patients); insomnia (4 [1%] patients); tremor (4 [1%] patients); and asthenia, akathisia, depression, sedation, irritability (each in 3 [1%] patients). Of the 41 (12%) patients who had an AE causing discontinuation, 14 had an AE that was considered related to study drug; the most common were psychiatric disorders (11 [3%] patients), nervous system disorders (8 [2%] patients), and cardiac disorders (7 [2%] patients). A total of 7 (2%) patients reported an AE of parkinsonism, with an EAIR of 0.01 patients per patient-year. All parkinsonism AEs were either mild or moderate in severity and generally resolved following dose reduction. One patient (<1%) discontinued from the study due to an AE of parkinsonism that was considered moderate in severity and probably related to study drug.

Pertinent safety scales measuring akathisia, anxiety, depression, sleepiness, and cognitive impairment generally remained unchanged over time (Supplementary Table 5). The mean change (SE) from baseline in UPDRS motor score was 0.5 (0.70) at Week 145, suggesting there were no notable changes in parkinsonism during the study. At baseline, 116 (34%) patients reported a history of mood disorders, including bipolar disorder or depression. In the separate assessment of suicidality using the C-SSRS, at screening, 82 (24%) patients reported suicidal ideation prior to the study, 61 (18%) patients reported suicidal behavior, and 11 (3%) patients reported self-injurious behavior without suicidal intent; at any time post-baseline, 22 (7%) patients displayed suicidal ideation, 2 (<1%) patients displayed suicidal behavior, and no patients displayed self-injurious behavior without suicidal intent.

## Discussion

The current analysis demonstrates the long-term benefits of deutetrabenazine for patients with TD who remain on treatment for up to 3 years, representing the longest prospective assessment in TD for a VMAT2 inhibitor. Treatment with deutetrabenazine showed robust, clinically meaningful, sustained reductions in dyskinesia, with two-thirds of patients achieving ≥50% and ~40% of patients achieving ≥70% improvement in total motor AIMS score from baseline. Most patients achieved treatment success based on CGIC and PGIC assessments, and patient-reported outcomes suggest that long-term deutetrabenazine treatment was associated with improved quality of life. Deutetrabenazine treatment was generally well tolerated, with most AEs considered mild or moderate in severity and low incidences of AEs commonly observed in patients with TD and VMAT2 inhibitor class–related AEs, including depression and parkinsonism. UPDRS motor score generally remained unchanged over 3 years, suggesting there was no notable increase in parkinsonism over time. There were no new safety signals identified over the course of the study, including no signals for increased suicide, torsade de pointes/QT prolongation, parkinsonism, or depression. Results of the current study demonstrate the sustained efficacy and favorable safety profile of long-term deutetrabenazine treatment in patients with TD.

Findings from this OLE study further suggest that deutetrabenazine may provide increasing benefit over time without an increase in dose. There was a relatively rapid improvement in total motor AIMS scores during the 6-week dose titration period, followed by slower but continued improvement through Week 145 (Figure 2). In contrast, mean daily deutetrabenazine dose was relatively stable post-titration, increasing only minimally from Week 15 to Week 145 (Figure 1). Analysis of mean change from baseline in total motor AIMS scores in patients who completed 54, 106, and 145 weeks of treatment suggests that the observed increasing benefit in AIMS score over time was not due to selective dropout of poor responders.

Other efficacy analyses also reflected sustained improvement and suggested the possibility of increasing benefit over time. The percent of patients experiencing ≥50% improvement in AIMS score from baseline increased over time (Figure 3). Treatment success (defined as “much improved” or “very much improved”) was achieved by the majority of patients at Week 15 and increased by Week 145 on both the CGIC and the PGIC. Together with data showing improvements in patient-focused measures, including mCDQ-24 total score and subdomains and AIMS items 9 and 10, patients noticed improvements in abnormal movements and these led to measurably better quality of life outcomes.

The mechanisms underlying the progressive improvement we observed remain speculative. It is possible that long-term treatment with deutetrabenazine induces a “depriming” effect wherein persistent normalization of dopaminergic tone ameliorates the underlying pathophysiologic changes of TD. This may occur over time just as TD develops over time. However, we note that our observations come from an open-label study and this result must be confirmed in a long-term double-blind study.

Additional limitations of this study include those inherent to the long-term open-label study design, including lack of a control group. Missing data caused by patient dropout were not imputed or otherwise accounted for; however, the effects of this limitation are mitigated by the additional *post-hoc* analyses of Week 54, Week 106, and Week 145 completers. Although the study period was extended from the originally planned timeframe of 1 to 3 years to collect longer-term data, reconsent requirements may have affected discontinuation rates. Deutetrabenazine was approved by the FDA and commercialized during the study, which may have led to some participants dropping out of the study. In addition, although there was a small change in the proportion of patients on a DRA at Week 145 compared with baseline of the parent studies (66% vs 75%), we are unable to differentiate whether AEs were attributable to deutetrabenazine or DRAs, as many potential AEs overlap between these medications.

The chronic and disabling nature of TD (26, 27), which is unremitting in most patients, coupled with the limited availability of treatment options emphasizes the need for safe and effective long-term treatment for patients with TD. Guidelines from the American Psychiatric Association recommend using a VMAT2 inhibitor to treat TD if prevention of DRA exposure is not possible, as there is minimal evidence to support the efficacy of antipsychotic dose reduction, and the potential benefit of dose reduction must be weighed against the risk of psychiatric relapse (14). Results from this OLE study demonstrate that long-term treatment with deutetrabenazine is associated with sustained improvements in efficacy and favorable safety and tolerability that persist over a period of 3 years and are clinically meaningful for patients, suggesting that deutetrabenazine may be a suitable option for patients with TD to manage their symptoms over time.

## Data Availability Statement

Qualified researchers may request access to patient-level data and related study documents including the study protocol and the statistical analysis plan. Requests will be reviewed for scientific merit, product approval status, and conflicts of interest. Patient-level data will be de-identified, and study documents will be redacted to protect the privacy of trial participants and to protect commercially confidential information. Please email USMedInfo@tevapharm.com to make your request.

## Ethics Statement

The studies involving human participants were reviewed and approved by Independent Ethics Committees and Institutional Review Boards at each site. A listing of all committees is provided as Supplementary Material. The patients/participants provided their written informed consent to participate in this study.

## Author Contributions

RH, HF, SF, JJ-S, and KA contributed to the design and conduct of the study, interpretation of the data, and drafting of the manuscript. HB and NG contributed to the analysis and interpretation of the data and drafting of the manuscript. LM, AW, JA, MG, and J-MS contributed to the design of the study, interpretation of the data, and drafting of the manuscript. RH accepts full responsibility for the finished work and/or the conduct of the study, had access to the data, and controlled the decision to publish. All authors reviewed and approved the final version of the manuscript for submission.

## Funding

This study was funded by Teva Pharmaceutical Industries Ltd., Tel Aviv, Israel (no award/grant number).

## Conflict of Interest

RH has served as a consultant for Teva Pharmaceutical Industries, AbbVie Inc., Acorda Therapeutics, Adamas Pharmaceuticals, AstraZeneca, Biotie Therapies, Cynapsus Therapeutics, Impax Laboratories, Inc., Lundbeck LLC, the Michael J. Fox Foundation, Neurocrine Biosciences, Neuropore Therapies, Pfizer Inc., Prexton Therapeutics, US WorldMeds, Guidepoint Global, Gerson Lehrman Group, LCN Consulting, Putnam Associates, National Parkinson Foundation, eResearch Technology, Inc., Sarepta Therapeutics, Back Bay Life Science, National Institutes of Health (NIH), Projects in Knowledge, Vista Research, LifeMax, PeerView Press, ClinicalMind Medical and Therapeutic Communications, Sunovion Pharmaceuticals, Academy for Continued Healthcare Learning, Outcomes Insights, Expert Connect, HealthLOGIX, Cowen and Company, Pharma Two B Ltd., RMEI Medical Education for Better Outcomes, ClearView Healthcare Partners, Health Advances, Kyowa Kirin Pharmaceutical Development, Inc., Quintiles, and Eli Lilly and Company. HB is an employee of Teva Pharmaceutical Industries. HF has received honoraria from Prime Education, Inc., International Parkinson and Movement Disorders Society, Carling Communications, Medscape (speaker in CME events), AbbVie Inc., Biogen, GE Healthcare, inVentiv, Kyowa Hakko Kirin, Lundbeck LLC, Merz Pharmaceuticals, Voyager, Sunovion Pharmaceuticals, and Pfizer Inc. (as a consultant); grant and research support from AbbVie Inc., Acadia, Teva Pharmaceutical Industries, Biotie Therapies/Acorda Therapeutics, Civitas, Kyowa/ProStrakan, the Michael J. Fox Foundation, International Parkinson and Movement Disorders Society, NIH/NINDS, Parkinson Study Group, Rhythm, and Synosia; royalties from Demos Publishing (serving as a book author/editor); and a stipend from the International Parkinson and Movement Disorders Society (MDS) for serving as a medical editor of the MDS website. The Cleveland Clinic has a contract with Teva Pharmaceutical Industries for Dr. Fernandez's role as a co-principal investigator in SD-809 tardive dyskinesia global studies. HF also serves as a member of the publication committee for Acorda Therapeutics but does not receive any personal compensation for this work. HF has no owner interest in any pharmaceutical company. SF has received honoraria from Neurocrine Biosciences, Lundbeck LLC, Teva Pharmaceutical Industries, Avanir, UCB, US WorldMeds, Sunovion Pharmaceuticals, and Adamas Pharmaceuticals; research support from Ipsen, Medtronic, Auspex, US WorldMeds, Pharm-Olam, Cynapsus Therapeutics/Sunovion Pharmaceuticals, Vaccinex, Solstice, CHDI Foundation, the Michael J. Fox Foundation, and the NIH; and royalties from Demos and Blackwell Futura. JJ-S has received consulting fees from Medtronic, St. Jude Medical, Boston Scientific, Blue Rock Therapeutics, Bracket Global, Teva, Nuvelution, CNS Ratings, AbbVie, Spark Therapeutics, Revance, Amneal, and Impel. NG is an employee of Teva Pharmaceutical Industries. LM is an employee of Teva Pharmaceutical Industries. AW is a former employee of Teva Pharmaceutical Industries. JA is a former employee of Teva Pharmaceutical Industries. MG is an employee of Teva Pharmaceutical Industries. J-MS is a former employee of Teva Pharmaceutical Industries. KA has served as a scientific advisor for LEGATO-HD, AIM-TD, ARM-TD, ENROLL-HD, and Prana; site principal investigator for PRIDE-HD, First-HD, ARC-HD, ENROLL-HD, and Vaccinex; and consultant for the NeuroNEXT 105 study. KA has received salary support from the Griffin Foundation and honoraria from Vindico Medical Education. The authors declare that this study received funding from Teva Pharmaceutical Industries Ltd., Tel Aviv, Israel. The funder had the following involvement in the study: design, collection of data, analysis and interpretation of the data, review and drafting of the manuscript.

## Publisher's Note

All claims expressed in this article are solely those of the authors and do not necessarily represent those of their affiliated organizations, or those of the publisher, the editors and the reviewers. Any product that may be evaluated in this article, or claim that may be made by its manufacturer, is not guaranteed or endorsed by the publisher.
