# Supplementary material for: Long-Term Deutetrabenazine Treatment for Tardive Dyskinesia Is Associated With Sustained Benefits and Safety: A 3-Year, Open-Label Extension Study
Source: Front Neurol. 2022 Feb 23;13:773999. doi: 10.3389/fneur.2022.773999 (PMC8906841; doi:10.3389/fneur.2022.773999)
Supplement: Supplementary file 1 [file Data_Sheet_1.docx]

**SUPPLEMENTARY MATERIALS**

**Supplemental Table 1. Full Study Inclusion and Exclusion Criteria Assessed at Screening in the Parent Studies, Unless Otherwise Specified.**

| **Inclusion criteria** |
| --- |
| - - Patient was ≥18 years of age at screening   - Patient had successfully completed AIM-TD, ARM-TD, or any other controlled study of deutetrabenazine for treatment of moderate to severe tardive dyskinesia (TD)   - Patient had a history of using a dopamine-receptor antagonist for ≥3 months (or 1 month in patients 60 years of age and older)   - Patient had a clinical diagnosis of TD and has had symptoms for ≥3 months prior to screening   - For patients with underlying psychiatric illness: - Patient was psychiatrically stable and had no change in psychoactive medications (including, but not limited to, antipsychotics, benzodiazepines, anticonvulsants, and mood stabilizers) for ≥30 days before screening (45 days for antidepressants) - Patients on long-acting (depot) medications have been on stable therapy (dose, frequency) ≥3 months before screening - Patient had a health care provider (caregiver) who was aware of the patient’s participation in the trial and did not anticipate any changes in the patient’s treatment regimen (drug, dose, frequency) in the next 3 months - Patient had a history of being compliant with prescribed medications - Patient was able to swallow study drug whole - Patient provided written informed consent or, if patient lacked the capacity to provide written informed consent, a legally authorized representative provided written informed consent and the patient provided assent - In the opinion of the investigator, the patient lived in a stable environment and had adequate supervision when necessary to comply with all study procedures, attend all study visits, and safely participate in the trial - Patient had sufficient reading skills to comprehend the patient-completed rating scales - Female patients of childbearing potential agreed to use one of the following acceptable methods of contraception from screening through study completion if sexually active:   - An intrauterine device or intrauterine system in place for ≥3 months prior to screening   - Patient or partner used barrier method (e.g., condom, diaphragm, or cervical cap) with spermicide from screening through study completion   - Partner had a documented vasectomy >6 months prior to enrollment   - Stable hormonal contraception (with approved oral, transdermal, or depot regimen) for ≥3 months prior to screening |
| **Exclusion criteria** |
| - - Patient had received tetrabenazine within 7 days of baseline   - Patient had received any of the following medications within 30 days of baseline:   - Reserpine, α-methyl-p-tyrosine, botulinum toxin (within 3 months of baseline), and medications with strong anticholinergic activity (trihexyphenidyl, benztropine, orphenadrine, procyclidine, and biperiden)   - Metoclopramide, promethazine, and prochlorperazine   - Stimulants (i.e., methylphenidate, amphetamine/dextroamphetamine, lisdexamphetamine, etc.) or monoamine oxidase inhibitors   - Levodopa or dopamine agonists   - Patient had a neurologic condition other than TD that could interfere with assessing the severity of dyskinesias   - Patient had a serious untreated or undertreated psychiatric illness   - Patient had active suicidal ideation at baseline   - Patient had a history of any of the following within 6 months of baseline:   - Previous intent to act on suicidal ideation with a specific plan, irrespective of level of ambivalence at the time of suicidal thought   - Previous preparatory acts to commit suicide or suicidal behavior   - A previous actual, interrupted, or aborted suicide attempt   - Patient had a score ≥11 on the depression subscale of the Hospital Anxiety and Depression Scale (HADS) at baseline   - Patient was developmentally disabled or had evidence of dementia   - Patient had an unstable or serious medical illness at baseline   - Patient had history (within 3 months) or presence of violent behavior   - Patient had a Fridericia’s corrected QT interval (QTcF) value >450 ms (males) or >460 ms (females), or >480 ms (with right bundle branch block) on 12-lead electrocardiogram (ECG) at baseline   - Patient had evidence of hepatic impairment at screening of the parent study, as indicated by:   - Aspartate transaminase (AST) or alanine aminotransferase (ALT) >2.5 times the upper limit of normal (ULN)   - Alkaline phosphatase (ALP) or total bilirubin (TBil) >2 times the ULN     - Note: Patients with Gilbert’s syndrome were eligible to participate if approved by the medical monitor     - Note: Patients with abnormalities in 2 or more of these analytes (AST, ALT, ALP, TBil) were to be approved by the medical monitor to be enrolled   - Prothrombin time >17 seconds (i.e., prothrombin time prolonged >4 seconds over the ULN   - Positive hepatitis B surface antigen   - Patient had evidence of significant renal impairment at screening of the parent study, indicated by a creatinine clearance <50 mL/min, as estimated by the Cockcroft-Gault formula   - Patient had known allergy to tetrabenazine or to any of the components of the study drug   - Patient had participated in an investigational drug or device trial (other than AIM-TD, ARM-TD, or any other eligible deutetrabenazine parent study) and received study drug within 30 days (or 5 drug half-lives) of baseline, whichever was longer   - Patient was pregnant or breastfeeding at baseline   - Patient acknowledged present use of illicit drugs at screening   - Patient had a history of alcohol or substance abuse in the previous 12 months, as defined in the *Diagnostic and Statistical Manual of Mental Disorders, 5^th^ Edition*, or patient was unable to refrain from substance abuse throughout the study |

**Supplemental Table 2. Reasons for Discontinuation From Study.**

| **Time period,  n (%)** | **AE^a^** | **Lack of efficacy** | **Lost to follow-up** | **Withdrawal by subject (reason unspecified)** | **Other** | **Any** |
| --- | --- | --- | --- | --- | --- | --- |
| <15 weeks  (n = 337) | 9 (3) | 2 (1) | 6 (2) | 12 (4) | 1 (<1) | 31 (9)^b^ |
| 15-<54 weeks  (n = 306) | 11 (4) | 5 (2) | 7 (2) | 29 (9) | 2 (1) | 57 (19)^c^ |
| 54-<106 weeks  (n = 249) | 14 (6) | 2 (1) | 8 (3) | 20 (7) | 0 | 44 (18) |
| 106-<145 weeks  (n = 194) | 4 (2) | 0 | 3 (2) | 10 (5) | 1 (1) | 19 (10)^d^ |
| Overall (N = 337) | 41 (12) | 9 (3) | 24 (7) | 79 (23) | 5 (1)^e^ | 163 (48)^f^ |

AE, adverse event.

^a^Includes death.

^b^Includes 1 patient who discontinued due to noncompliance with study drug.

^c^Includes 2 patients who discontinued due to noncompliance with study drug and 1 patient who discontinued due to protocol deviation.

^d^Includes 1 patient who discontinued due to study terminated.

^e^Includes the following reasons: Patient chose on his own to stop taking medication sometime between study visits, subject does not wish to continue with the study, site closing, principal investigator decision, and subject no longer lives in the area and does not want to travel to the site.

^f^Includes 3 patients who discontinued due to noncompliance with study drug, 1 patient who discontinued due to protocol deviation, and 1 patient who discontinued due to study termination.

**Supplemental Table 3. Baseline Demographic and Clinical Characteristics^a^.**

|  | **All patients (N = 337)** |
| --- | --- |
| **Patient demographics** |  |
| Age (years), mean (SD) | 56.9 (10.65) |
| Sex, female, n (%) | 188 (56) |
| Race, White, n (%) | 264 (78) |
| Weight (kg), mean (SD) | 82.6 (20.49) |
| BMI (kg/m^2^), mean (SD) | 29.23 (6.98) |
| **Patient clinical characteristics** |  |
| Total motor AIMS score (items 1–7), mean (SD) | 10.7 (4.68) |
| TD duration (years), mean (SD) | 5.7 (5.75) |
| Receiving DRA at baseline, n (%) | 254 (75) |
| **Background comorbid illness** |  |
| Psychotic disorders,^b^ n (%) | 205 (61) |
| Mood disorders,^c^ n (%) | 116 (34) |
| Other or missing, n (%) | 16 (5) |

SD, standard deviation; BMI, body mass index; AIMS, Abnormal Involuntary Movement Scale; TD, tardive dyskinesia; DRA, dopamine-receptor antagonist; OLE, open-label extension.
^a^Baseline characteristics were recorded at the start of each pivotal study, except baseline total motor AIMS score, which was recorded at the start of the OLE study.

^b^Schizophrenia, schizoaffective disorder.
^c^Bipolar disorder, depression.

**Supplemental Table 4. Abnormal Postbaseline QTcF Values^a.^**

| **QTcF category, n (%)** | **All patients (N = 337)** |
| --- | --- |
| >450 ms | 30 (9) |
| >480 ms | 6 (2) |
| >500 ms | 4 (1) |
| Change >30 ms | 45 (13) |
| Change >60 ms | 5 (1) |

QTcF, Fridericia’s corrected QT interval.

^a^Patients were counted only once in each category.

**Supplemental Table 5. Mean Change From Baseline in Safety Scales.**

| **Safety assessment scale,  mean change (SE) from baseline** | **Timepoint** | | | |
| --- | --- | --- | --- | --- |
|  | **Week 54** | **Week 106** | **Week 132** | **Week 145** |
| UPDRS | 0.1 (0.40) | 0.8 (0.61) | 0.4 (0.65) | 0.5 (0.70) |
| BARS | –0.4 (0.10) | –0.4 (0.13) | –0.6 (0.14) | –0.7 (0.14) |
| HADS anxiety total | –0.1 (0.23) | –0.1 (0.26) | –0.2 (0.27) | –0.3 (0.27) |
| HADS depression total | 0.6 (0.23) | 0.4 (0.27) | 0.4 (0.26) | 0.5 (0.27) |
| ESS | 0.1 (0.21) | –0.1 (0.25) | 0.0 (0.27) | – |
| MoCA | 0.0 (0.15) | –0.2 (0.16) | –0.5 (0.20) | – |

SE, standard error; UPDRS, Unified Parkinson’s Disease Rating Scale; BARS, Barnes Akathisia Rating Scale; HADS, Hospital Anxiety and Depression Scale; ESS, Epworth Sleepiness Scale; MoCA; Montréal Cognitive Assessment.

**Supplemental Figure 1. Study design^a^.**


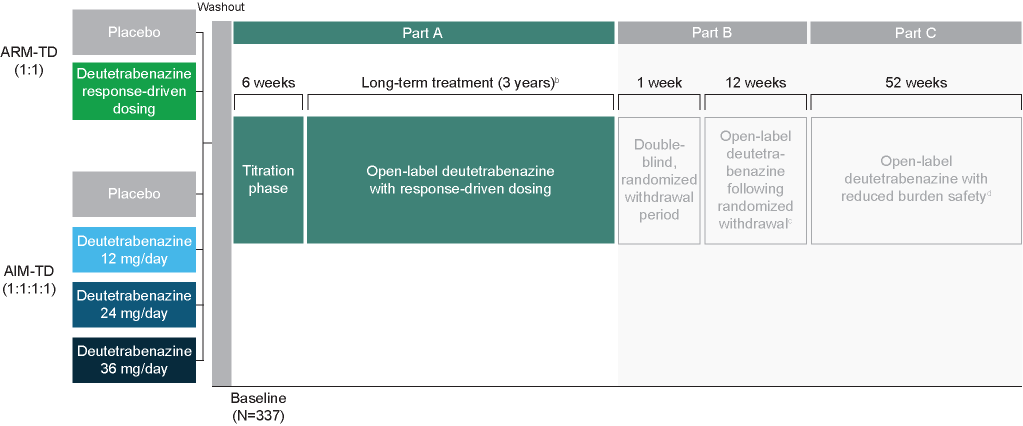


ET, early termination.

^a^Data in this report are included for Part A only. ^b^Patient participation in Part A ended at Week 158, at the start of Part B, or ET. Patients were on a stable dose of deutetrabenazine and any concomitant dopamine-receptor antagonist for a minimum of 4 weeks before starting the double-blind, randomized withdrawal period (Part B).

^c^Following the 1-week double-blind, randomized withdrawal period, patients in Part B were returned to their previous deutetrabenazine dose.

^d^Patients in European Union countries in Part C continued deutetrabenazine treatment at the dose administered during the 12-week open-label period of Part B.

**Supplemental Figure 2. Patient disposition.**

**
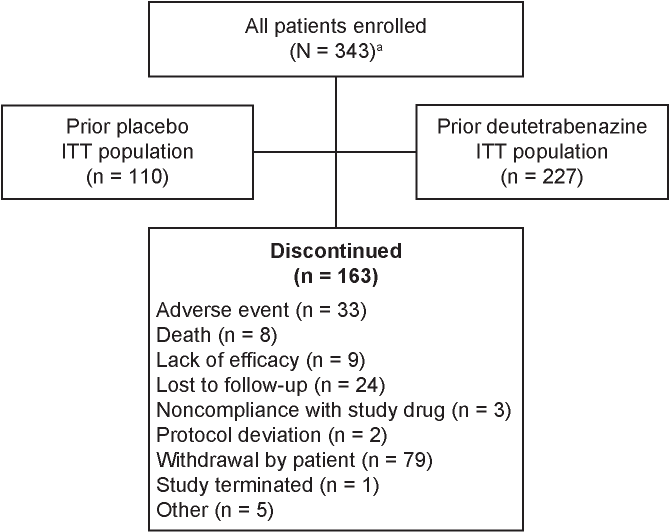
**

ITT, intent-to-treat.

^a^1 site (6 patients) was excluded from all analyses due to site data integrity issues.

**Supplemental Figure 3. Mean change from baseline in mCDQ-24 subdomain scores over time.**


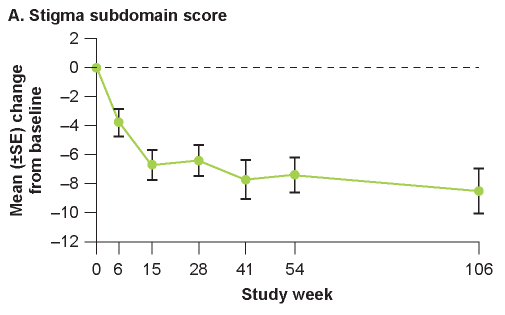


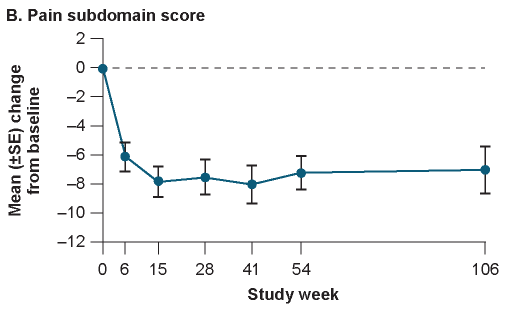


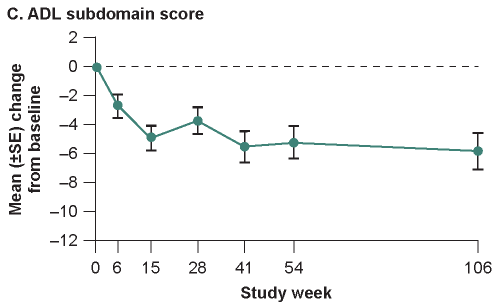


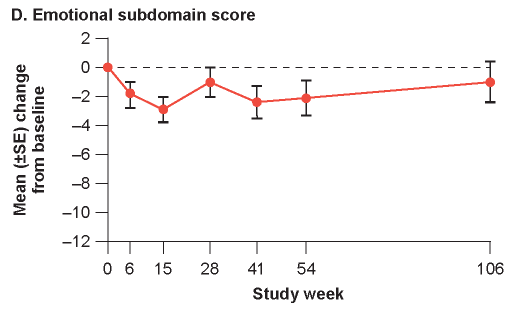


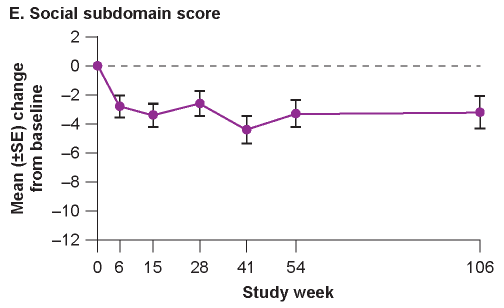


mCDQ-24, modified Craniocervical Dystonia Questionnaire; SE, standard error; ADL, activities of daily living.
