# Supplementary material for: Long-Term Deutetrabenazine Treatment for Tardive Dyskinesia Is Associated With Sustained Benefits and Safety: A 3-Year, Open-Label Extension Study
Source: Front Neurol. 2022 Feb 23;13:773999. doi: 10.3389/fneur.2022.773999 (PMC8906841; doi:10.3389/fneur.2022.773999)
Supplement: Supplementary file 2 [file Data_Sheet_2.docx]

**List of Independent Ethics Committees and Institutional Review Boards (Study Centers Where Patients Were Screened)**

| **Center number** | **IEC/IRB name** | **Country** |
| --- | --- | --- |
| 101 | Advarra  Formerly: Chesapeake IRB | USA |
| 167 | Advarra  Formerly: Chesapeake IRB | USA |
| 169 | Advarra  Formerly: Chesapeake IRB | USA |
| 123 | Advarra  Formerly: Chesapeake IRB | USA |
| 162 | Advarra  Formerly: Chesapeake IRB | USA |
| 178 | Advarra  Formerly: Chesapeake IRB | USA |
| 104 | Advarra  Formerly: Chesapeake IRB | USA |
| 144 | Advarra  Formerly: Chesapeake IRB | USA |
| 161 | Advarra  Formerly: Chesapeake IRB | USA |
| 157 | Advarra  Formerly: Chesapeake IRB | USA |
| 146 | Advarra  Formerly: Chesapeake IRB | USA |
| 160 | Advarra  Formerly: Chesapeake IRB | USA |
| 174 | Advarra  Formerly: Chesapeake IRB | USA |
| 129 | Advarra  Formerly: Chesapeake IRB | USA |
| 107 | Advarra  Formerly: Chesapeake IRB | USA |
| 151 | Advarra  Formerly: Chesapeake IRB | USA |

| **Center number** | **IEC/IRB name** | **Country** |
| --- | --- | --- |
| 110 | Advarra  Formerly: Chesapeake IRB | USA |
| 153 | Advarra  Formerly: Chesapeake IRB | USA |
| 150 | Advarra  Formerly: Chesapeake IRB | USA |
| 112 | Advarra  Formerly: Chesapeake IRB | USA |
| 114 | Advarra  Formerly: Chesapeake IRB | USA |
| 115 | Advarra  Formerly: Chesapeake IRB | USA |
| 117 | Advarra  Formerly: Chesapeake IRB | USA |
| 102 | Advarra  Formerly: Chesapeake IRB | USA |
| 118 | Advarra  Formerly: Chesapeake IRB | USA |
| 130 | Advarra  Formerly: Chesapeake IRB | USA |
| 108 | Advarra  Formerly: Chesapeake IRB | USA |
| 156 | Georgetown University Institutional Review Board | USA |
| 175 | Saint Louis University Institutional Review Board | USA |
| 165 | Emory University IRB | USA |
| 128 | University of New Mexico HSC Human Research Review Committee | USA |

| **Center number** | **IEC/IRB name** | **Country** |
| --- | --- | --- |
| 149 | University of Tennessee HSC IRB | USA |
| 155 | Western Institutional Review Board | USA |
| 131 | Northwestern University Institutional Review Board Biomedical IRB | USA |
| 154 | Johns Hopkins Medicine  Office of Human Subjects Research Institutional Review Boards | USA |
| 145 | Tuscaloosa VAMC Subcommittee on Human Studies | USA |
| 142 | Institutional Review Board University of Missouri-Kansas City | USA |
| 121 | University of Southern California Institutional Review Board | USA |
| 502 | **Coordinating EC:**  Ethik-Kommission der Landesärztekammer Baden- Württemberg  **Local EC:**  Ethik-Kommission der Landesärztekammer Thüringen | Germany |
| 504 | **Coordinating EC:**  Ethik-Kommission der Landesärztekammer Baden- Württemberg  **Local EC:**  Ethikkommission der Landesärztekammer Rheinland Pfalz | Germany |
| 508 | **Local EC and coordinating EC:** Komisja Bioetyczna przy Okręgowej Izbie Lekarskiej w  Łodzi | Poland |

| **Center number** | **IEC/IRB name** | **Country** |
| --- | --- | --- |
| 509 | **Coordinating EC:** Komisja Bioetyczna przy Okręgowej Izbie Lekarskiej w Łodzi  **LEC:** Komisja Bioetyczna przy Okręgowej Izbie Lekarskiej w Krakowie | Poland |
| 510 | **Coordinating EC:** Komisja Bioetyczna przy Okręgowej Izbie Lekarskiej w Łodzi  **LEC**: Komisja Bioetyczna przy Okręgowej Izbie Lekarskiej w Bydgoszczy | Poland |
| 511 | **Coordinating EC:** Komisja Bioetyczna przy Okręgowej Izbie Lekarskiej w Łodzi  **LEC:** Komisja Bioetyczna przy Okręgowej Izbie Lekarskiej w Lublinie | Poland |
| 512 | **Coordinating EC:** Komisja Bioetyczna przy Okręgowej Izbie Lekarskiej w Łodzi  **LEC:** Komisja Bioetyczna Śląskiej Izby Lekarskiej w Katowicach | Poland |
| 513 | **Coordinating EC:** Komisja Bioetyczna przy Okręgowej Izbie Lekarskiej w Łodzi  **LEC:** Niezależna Komisja Bioetyczna przy Gdańskim  Uniwersytecie Medycznym | Poland |
| 514 | **LEC and Coordinating EC:** Komisja Bioetyczna przy Okręgowej Izbie Lekarskiej w  Łodzi | Poland |
| 516 | **Coordinating EC:** Komisja Bioetyczna przy Okręgowej Izbie Lekarskiej w Łodzi  **LEC:** Komisja Bioetyczna przy Dolnośląskiej Izbie Lekarskiej | Poland |
| 517 | **Coordinating EC:** Komisja Bioetyczna przy Okręgowej Izbie Lekarskiej w Łodzi  **LEC:** Komisja Bioetyczna przy Okręgowej Izbie Lekarskiej w Białymstoku | Poland |

| **Center number** | **IEC/IRB name** | **Country** |
| --- | --- | --- |
| 519 | **Coordinating EC:** Komisja Bioetyczna przy Okręgowej Izbie Lekarskiej w Łodzi  **LEC:** Komisja Bioetyczna przy Bydgoskiej Izbie Lekarskiej | Poland |
| 520 | **Coordinating EC:** Komisja Bioetyczna przy Okręgowej Izbie Lekarskiej w Łodzi  **LEC:** Komisja Bioetyczna przy Okręgowej Izbie Lekarskiej w  Krakowie | Poland |
| 522 | **Coordinating EC:** Komisja Bioetyczna przy Okręgowej Izbie Lekarskiej w Łodzi  **LEC:** Komisja Bioetyczna przy  Kujawsko-Pomorskiej Okręgowej Izbie Lekarskiej w Toruniu | Poland |
| 523 | **Coordinating EC:** Komisja Bioetyczna przy Okręgowej Izbie Lekarskiej w Łodzi  **LEC:** Komisja Bioetyczna przy Kujawsko-Pomorskiej Okręgowej Izbie Lekarskiej w Toruniu | Poland |
| 526 | MEC: Etická komisia, Psychiatrická nemocnica Hronovce  **LEC:** Etická komisia, Nemocnica s poliklinikou sv. Barbory Rožňava, a.s., | Slovakia |
| 527 | MEC: Etická komisia, Psychiatrická nemocnica Hronovce  **LEC:** Etická komisia KSK | Slovakia |
| 529 | **MEC:** Etická komisia, Psychiatrická nemocnica Hronovce  **LEC:** Etická komisia BSK | Slovakia |
| 530 | **MEC: + LEC:**  Etická komise Fakultni nemocnice Motol | Czech Republic |

| **Center number** | **IEC/IRB name** | **Country** |
| --- | --- | --- |
| 533 | **MEC:** Etická komise Fakultni nemocnice Motol  **LEC:** Eticka komise Clintrial s.r.o. | Czech Republic |
| 535 | **MEC:** Etická komise Fakultni nemocnice Motol  **LEC:** Eticka komise BIALBI s.r.o. | Czech Republic |
| 538 | **MEC:**  Egészségügyi Tudományos Tanács  Klinikai Farmakológiai Etikai Bizottság,  **LEC:**  Nyirő Gyula Kórház-OPAl Intezményi Kutatásetikai Bizottság | Hungary |
| 539 | **MEC:**  Egészségügyi Tudományos Tanács  Klinikai Farmakológiai Etikai Bizottság,  **LEC:**  Veszprém Megyei Csolnoky Ferenc Kórház  Intezményi Kutatásetikai Bizottság | Hungary |
| 540 | **MEC:**  Egészségügyi Tudományos Tanács  Klinikai Farmakológiai Etikai Bizottság  **LEC:**  Dr. Kenessey Albert Kórház es Rendelőintézet  Intezményi Kutatásetikai Bizottság | Hungary |
| 541 | **MEC:**  Egészségügyi Tudományos Tanács  Klinikai Farmakológiai Etikai Bizottság  **LEC:**  Semmelweis Egyetem Igazságügyi Orvostani Intézet  Regonális, Intézményi Tudományos és Kutatásetikai Bizottság | Hungary |

| **Center number** | **IEC/IRB name** | **Country** |
| --- | --- | --- |
| 545 | **MEC:**  Egészségügyi Tudományos Tanács  Klinikai Farmakológiai Etikai Bizottság  **LEC:**  Semmelweis Halasi Kórház Intezményi Kutatásetikai Bizottság, | Hungary |
| 546 | **MEC:**  Egészségügyi Tudományos Tanács  Klinikai Farmakológiai Etikai Bizottság  **LEC:**  Petz Aladár Megyei Oktató Kórház Intezményi Kutatásetikai Bizottság | Hungary |
| 549 | **Coordinating EC:** Komisja Bioetyczna przy Okręgowej Izbie Lekarskiej w Łodzi  **LEC:** Komisja Bioetyczna przy Warmińsko-Mazurskiej Izbie  Lekarskiej | Poland |
| 550 | **Coordinating EC:** Komisja Bioetyczna przy Okręgowej Izbie Lekarskiej w Łodzi  **LEC:** Komisja Bioetyczna Okręgowej Izby Lekarskiej w  Warszawie | Poland |
| 554 | **Coordinating EC:** Komisja Bioetyczna przy Okręgowej Izbie Lekarskiej w Łodzi  **LEC:** Komisja Bioetyczna przy Okręgowej Izbie Lekarskiej w  Białymstoku | Poland |
| 556 | **MEC: + LEC:**  Etická komise Fakultni nemocnice Motol | Czech Republic |
| 557 | **MEC:** Etická komise Fakultni nemocnice Motol  **LEC:** Eticka komise nestatniho zdravotnickeho zarizeni Research Site s.r.o. | Czech Republic |
| 559 | **MEC: + LEC:**  Etická komise Fakultni nemocnice Motol | Czech Republic |
